# Myoprotective Fat-Loss Phenotypes in Obesity-Associated Type 2 Diabetes: A 12-Month Real-World Cohort Study of Metformin-Based Treatment Regimens

**DOI:** 10.3390/clinpract16080141

**Published:** 2026-07-28

**Authors:** Ioana Bujdei-Tebeică, Anca Mihaela Pantea-Stoian, Doina Andrada Mihai, Simona Diana Ștefan, Cristian Serafinceanu

**Affiliations:** 1 Department of Diabetes, Nutrition and Metabolic Diseases, Carol Davila University of Medicine and Pharmacy, 050474 Bucharest, Romania; 2 National Institute of Diabetes, Nutrition and Metabolic Diseases N.C. Paulescu, 020475 Bucharest, Romania

**Keywords:** type 2 diabetes mellitus, obesity, fat-mass reduction, lean-mass preservation, skeletal muscle mass, handgrip strength, sarcopenic obesity, real-world cohort

## Abstract

**Background/Objectives**: In obesity-associated type 2 diabetes (T2DM), therapeutic benefit increasingly requires assessment of not only HbA1c and total body weight, but also the composition and functional quality of weight change. We evaluated a myoprotective fat-loss phenotype, defined as a reduction in adiposity accompanied by preservation of lean mass and handgrip strength. **Methods**: This secondary patient-level analysis included 166 adults with T2DM who completed 12 months of follow-up without changing treatment in a real-world tertiary diabetes cohort. Patients received metformin alone or metformin combined with a sulfonylurea, a DPP-4 inhibitor, an SGLT2 inhibitor, a GLP-1 receptor agonist, or insulin. Body composition was assessed by bioimpedance and muscle function by handgrip dynamometry. The primary phenotype required a reduction in fat mass ≥5%, a loss of lean mass <3%, and a decrease in handgrip ≤1 kg. Phenotype distributions were compared between treatment groups; patient-level associations and adjusted contrasts were explored using correlation analyses and baseline-adjusted regression models with robust HC3 standard errors. **Results**: Fat-mass reduction ≥5% occurred in 58/166 patients (34.9%), lean-mass preservation in 129/166 (77.7%), and handgrip preservation in 143/166 (86.1%). The complete myoprotective phenotype was present in 35/166 patients (21.1%) and differed significantly across treatment groups (χ^2^ = 131.58; permutation *p* < 0.0001). It was most frequent in the GLP-1 receptor agonist group (13/23; 56.5%) and the SGLT2 inhibitor group (12/25; 48.0%), less frequent with metformin monotherapy (9/46; 19.6%) and DPP-4 inhibitors (1/17; 5.9%), and absent in the sulfonylurea and insulin groups. GLP-1 receptor agonists showed the greatest crude fat-mass loss, whereas lean-mass loss, skeletal-muscle-mass change, and handgrip change did not differ significantly across groups after false-discovery-rate correction. **Conclusions**: Myoprotective fat-loss phenotypes can be identified in obesity-associated T2DM using body composition and handgrip measures. These observational findings support the assessment of the quality, not just the magnitude, of weight loss in diabetes care and require validation in larger prospective studies.

## 1. Introduction

Type 2 diabetes mellitus (T2DM) is no longer considered a disorder defined exclusively by chronic hyperglycemia. Although HbA1c remains essential for diagnosis, monitoring, and risk stratification, long-term prospective data have shown a graded association between glycemic exposure and diabetes-related outcomes, including microvascular complications [1]. Contemporary diabetes care increasingly recognizes T2DM as a cardio-reno-metabolic disease in which glycemic dysregulation interacts with obesity, ectopic fat accumulation, insulin resistance, inflammation, renal vulnerability, cardiovascular risk, and progressive functional decline [2,3]. This broader framework is particularly relevant in obesity-associated T2DM, where excess dysfunctional adipose tissue is not only a comorbidity but also a biological driver of insulin resistance, beta-cell stress, low-grade inflammation, dyslipidemia, hypertension, and long-term cardiometabolic risk [3,4,5,6].

Therefore, therapeutic evaluation in T2DM has progressively shifted from a predominantly glucose-centered model to a multidimensional model that includes body weight, adiposity, cardiorenal risk, hypoglycemia risk, treatment tolerability, and patient-centered outcomes [7]. Current standards of care emphasize weight management as a fundamental component of diabetes prevention and treatment, especially in overweight or obese patients [8]. However, total body weight is an aggregate measure. It does not distinguish fat-mass reduction from lean-mass loss, changes in skeletal muscle mass, fluid changes, or preservation of functional capacity. Therefore, two patients with comparable weight loss may have substantially different biological and clinical responses [9].

This distinction has become increasingly important in the current therapeutic era. Glucagon-like peptide-1 receptor agonists (GLP-1 RAs), dual incretin-based therapies, and sodium-glucose cotransporter-2 inhibitors (SGLT2i) have expanded the goals of diabetes treatment beyond glycemic control alone, with clinically relevant effects on body weight, adiposity, and cardio-reno-metabolic outcomes [10,11,12]. However, pharmacologically induced weight loss raises an important question regarding body composition: is the weight loss predominantly attributable to adipose tissue loss or does it include clinically relevant reductions in lean mass and skeletal muscle mass? The recent literature on GLP-1 RA and dual incretin therapies suggests that fat loss generally predominates, but lean-mass reductions may also occur, with variations across populations, interventions, and assessment methods [10,11,12,13]. Similarly, studies of SGLT2 inhibitors suggest favorable effects on body weight, waist circumference, and fat mass, although potential effects on fat-free mass, skeletal muscle mass, and risk of sarcopenia remain an area of ongoing investigation [14].

The clinical relevance of this issue is amplified by the close relationship between T2DM, obesity, and impaired muscle health. Skeletal muscle is a major site of insulin-mediated glucose disposal and plays a central role in metabolic flexibility, physical performance, and functional independence [15,16]. In patients with T2DM, insulin resistance, chronic inflammation, ectopic lipid accumulation, mitochondrial dysfunction, oxidative stress, neuropathy, vascular disease, and reduced physical activity may contribute to the deterioration of muscle quantity, muscle quality, and muscle function [16,17,18]. When excess adiposity coexists with reduced muscle mass or function, the phenotype may overlap with sarcopenic obesity, a condition associated with impaired mobility, risk of frailty, cardiometabolic burden, and poorer clinical outcomes [17,18,19]. Therefore, in obesity-associated T2DM, a strategy that reduces weight but compromises muscle reserve may be less favorable than one that selectively reduces adiposity while preserving muscle mass and strength [18,19].

For this reason, the quality of weight loss deserves special attention. From a functional and metabolic perspective, the most desirable pattern is not weight loss per se, but a reduction in adiposity accompanied by preservation of lean mass, skeletal muscle mass, and muscle function [20,21,22]. In contrast, weight loss accompanied by a disproportionate loss of lean mass or a decrease in handgrip strength may indicate a potentially unfavorable or incomplete response, especially in older patients, patients with long-standing diabetes, or patients with reduced functional reserve [21,22,23]. This distinction is clinically relevant because the same absolute reduction in body weight may represent either a myoprotective adiposity response or a mixed response with possible functional cost [20,23].

Body-composition assessment offers a practical way to go beyond the limitations of body weight and BMI. Bioelectrical impedance analysis allows the estimation of fat mass, fat-free mass, total body water, and indices derived from muscle activity in routine clinical settings, provided that standardized procedures and appropriate equations are used [24]. In T2DM, bioelectrical impedance analysis has been specifically discussed as a feasible approach for assessing body composition in patients with or at risk of sarcopenia [25]. Although imaging-based techniques such as dual-energy X-ray absorptiometry remain the gold standard for detailed compartmental analysis, recent validation data in older adults with T2DM support the clinical utility of multifrequency bioelectrical impedance analysis for assessing muscle mass when baseline and follow-up assessments are available [26]. In parallel, handgrip strength provides a simple and clinically interpretable measure of muscle function. The revised European consensus on sarcopenia emphasizes reduced muscle strength as a key feature of sarcopenia and recognizes muscle strength as a highly relevant marker of muscle function [27]. Thus, combining body composition assessment with handgrip dynamometry may help characterize whether weight loss is functionally favorable or potentially myopenic [25,26,27].

Despite the growing interest in weight-focused diabetes care, most clinical assessments still report mean changes in HbA1c, body weight, or BMI, while less attention is paid to the composition and functional consequences of weight change [28,29]. This creates an evidence gap in routine clinical practice. Reduction in fat mass without deterioration in lean mass or handgrip strength may identify a high-quality response phenotype, whereas reduction in fat mass accompanied by loss of lean mass or functional decline requires additional clinical interpretation [28]. Similarly, the lack of fat-mass reduction despite improvement in glycemia may suggest a discordant response in which the adiposity-driven component of the disease remains insufficiently modified. Such patient-level phenotyping can complement conventional treatment comparisons and support a more individualized interpretation of therapeutic response [29,30,31].

Real-world evidence is particularly important because patients encountered in routine diabetes care are often older, have longer diabetes duration, a higher burden of obesity, multimorbidity, variable adherence, and more heterogeneous treatment exposure than participants in randomized controlled trials [32,33]. Eastern European cohorts are less consistently represented or analyzed separately in many large cardiometabolic and obesity-focused clinical trials, despite a high regional burden of T2DM, obesity, and associated complications [34,35]. Therefore, real-world analyses that include anthropometry, bioimpedance-derived body composition, functional measurements, and longitudinal follow-up may provide clinically relevant information for diabetes care at the regional and broader levels [32,35].

The present study was designed as a patient-level secondary analysis of a 12-month cohort of adults with obesity-related T2DM treated with stable metformin-based antidiabetic regimens in the real world. The primary aim was to assess the functional quality of weight loss by identifying patients who achieved fat-mass reduction while maintaining lean mass and handgrip strength [20,21,22,23]. We defined a myoprotective fat-loss phenotype as the combined presence of a reduction in fat mass of at least 5%, a loss of lean mass of less than 3%, and the absence of a decrease in handgrip strength of more than 1 kg over a 12-month period. Secondary objectives were to describe the distribution of this phenotype across treatment classes, characterize individual body composition and functional-response measures, assess baseline differences by phenotype, assess treatment-adjusted differences in body composition and functional indices, and explore correlations between adiposity reduction and changes in handgrip strength.

We hypothesized that treatment response in obesity-associated T2DM would be heterogeneous and that clinically interpretable myoprotective and non-myoprotective fat-loss phenotypes could be identified [28,29,30,31]. Specifically, we expected that some patients would achieve fat loss without detectable impairment in lean muscle mass or handgrip strength, whereas others would exhibit either an inadequate fat response or patterns potentially associated with muscle or functional costs. By focusing on the quality of weight loss, rather than just its magnitude, this analysis aimed to support a more integrated interpretation of antidiabetic therapy in patients with obesity-associated T2DM.

## 2. Materials and Methods

### 2.1. Study Design and Setting

This study was designed as a patient-level secondary analysis of a prospective, real-world observational cohort of adults with T2DM and obesity-related metabolic diseases. The present analysis focused on the functional quality of weight loss, defined as adiposity reduction accompanied by preservation of lean mass and muscle function over a 12-month period of stable antidiabetic therapy.

The cohort was recruited and followed at the National Institute of Diabetes, Nutrition and Metabolic Diseases Prof. N.C. Paulescu, a tertiary referral center for diabetes mellitus in Bucharest, Romania. Baseline assessments were performed during routine outpatient care between June and December 2024, and 12-month follow-up assessments were completed between June and December 2025. Treatment allocation was not randomized and reflected routine clinical decision-making. Therefore, treatment-related comparisons were interpreted as observational associations rather than causal treatment effects. The study was reported in accordance with the STROBE guidelines for observational studies [36].

### 2.2. Study Population and Analytic Cohort

Adults with confirmed T2DM were assessed for eligibility. Patients were considered eligible if they were receiving metformin monotherapy or metformin-based therapy in combination with a predefined second-line antidiabetic class and had paired assessments at baseline and 12 months of follow-up. To allow interpretation of body composition and functional changes under stable pharmacological exposure, patients were required to remain on the same antidiabetic regimen throughout the 12-month observation period.

Exclusion criteria included complex or unclassifiable antidiabetic regimens, continuous triple antidiabetic therapy outside the predefined analytical framework, severe anemia, severe hypertriglyceridemia, duplicate records, missing baseline data, incomplete key body composition or functional variables, treatment change during follow-up, withdrawal from the study, loss of data during follow-up, or absence of assessment at 12 months.

Of the 291 adults with T2DM evaluated, 202 met the eligibility criteria. Of these, 166 patients completed a 12-month follow-up period without treatment change and had the necessary variables for this analysis of the myoprotective weight loss phenotype. These 166 patients constituted the final analytic cohort.

Of the 202 eligible patients, 36 were not retained in the analytic cohort. Because patients who required treatment intensification or switching were excluded by design, the analytic cohort is enriched for individuals whose therapy remained clinically adequate over 12 months. The resulting population is therefore metabolically more stable than the source clinic population, and the phenotype frequencies reported here should be read as applying to patients maintained on an unchanged metformin-based regimen rather than to all patients initiating these therapies. This restriction was necessary because the exposure of interest was sustained 12-month pharmacological exposure, which cannot be defined in patients whose regimen changes mid-observation; the trade-off is a conservative bias against detecting deterioration in body composition, since patients with the poorest metabolic trajectories are the most likely to have had therapy intensified and thus to have been excluded.

Adherence to antidiabetic therapy was not quantified using pill counts, pharmacy refill records, electronic monitoring, or a validated adherence questionnaire. Sustained treatment exposure was instead operationalized as continuation of the same prescribed regimen, verified against the prescription record and the treating physician’s notes at every scheduled outpatient visit across the 12-month period. Dose adjustment, treatment interruption, addition of a further agent, or switching between classes rendered a patient ineligible for the analytic cohort; consequently, no dose modifications occurred during follow-up among the 166 patients analyzed, and no dose-change term was required in the analysis. This approach establishes the persistence of prescribed exposure and stability of the prescribed dose, but it does not verify day-to-day medication intake, and residual non-adherence cannot be excluded.

The duration of the specific antidiabetic regimen before study entry was not systematically recorded and could therefore not be compared across treatment groups or entered as a covariate. Patients were receiving their regimen at the time of the baseline assessment rather than initiating it at enrolment, so the 12-month changes reported here reflect continued maintenance exposure rather than incident treatment initiation. This distinction matters for interpretation, because the body-composition changes that follow initiation of a GLP-1 receptor agonist or an SGLT2 inhibitor are typically largest in the first months of therapy; if pre-entry treatment duration differed systematically between groups, the between-group differences observed here could be attenuated or accentuated accordingly. Duration of diabetes, which was recorded, was included as a covariate in all adjusted models and serves as a partial proxy for cumulative treatment exposure.

All participants provided written informed consent. The study was conducted in accordance with the Declaration of Helsinki and was approved by the Ethics Committee of the Carol Davila University of Medicine and Pharmacy, Bucharest (approval number 15142, dated 3 June 2022) [37].

### 2.3. Treatment Exposure

The antidiabetic regimen maintained during the 12-month observation period was used as an exposure variable. Patients were classified into six mutually exclusive treatment groups:

1. Metformin monotherapy (*n* = 46);
2. Metformin plus sulfonylurea (*n* = 10);
3. Metformin plus dipeptidyl peptidase-4 inhibitor (*n* = 17);
4. Metformin plus sodium-glucose cotransporter-2 inhibitor (*n* = 25);
5. Metformin plus glucagon-like peptide-1 receptor agonist (*n* = 23);
6. Metformin plus insulin (*n* = 45).

Treatment class was used to describe the distribution of myoprotective and non-myoprotective fat-loss phenotypes across treatment regimens. Because treatment allocation was not randomized, the analysis was not intended to estimate causal comparative efficacy. The primary unit of interpretation was individual patient body composition and functional-response pattern, rather than isolated treatment mean contrasts.

Within each combination group, second-line agents were prescribed at fixed maintenance doses in accordance with national reimbursement criteria and institutional prescribing practice. SGLT2 inhibitor therapy consisted of dapagliflozin 10 mg or empagliflozin 10 mg once daily, and GLP-1 receptor agonist therapy consisted of semaglutide 1 mg once weekly or dulaglutide 1.5 mg once weekly. Dosing within these two groups was therefore effectively uniform, with no titration during the observation period, since any dose modification resulted in exclusion from the analytic cohort. This near-absence of dose variation is a design feature rather than an omission: it removes dose as a source of within-group heterogeneity and permits the observed phenotype distribution to be attributed to the class of agent under a stable maintenance dose, but it also means that dose–response relationships cannot be examined in this cohort.

Treatment regimen was defined as the exposure variable for the purpose of descriptive stratification and not for comparative effectiveness estimation. The regimen maintained unchanged over the full 12 months is the clinically meaningful stratum under which body-composition and functional trajectories are actually observed in routine care, and it corresponds to the therapeutic classes between which a clinician chooses. Stratifying by regimen therefore allows the distribution of response phenotypes to be characterized in the terms in which treatment decisions are made. The adjusted models reported in Section 3.5 were fitted to improve the precision of this description and to account for baseline imbalance in the corresponding outcome, age, sex, diabetes duration, and BMI; they were not fitted to support causal comparative claims, and no formal head-to-head hypothesis was prespecified. Adjusted treatment contrasts should accordingly be interpreted as baseline-adjusted descriptive differences between non-randomized strata, not as estimates of comparative treatment effect.

### 2.4. Clinical, Anthropometric, Functional, and Laboratory Assessments

Clinical, anthropometric, functional, and laboratory assessments were performed at baseline and after 12 months of follow-up. Baseline variables included age, sex, duration of diabetes, smoking, hypertension, cardiovascular disease, chronic kidney disease, dyslipidemia, obesity class, use of statins, use of angiotensin-converting enzyme inhibitors or angiotensin receptor blockers, recent infection, use of anti-inflammatory drugs, use of glucocorticoids, physical activity level, resistance training, and available adherence indicators.

Anthropometric assessment included body weight, height, body mass index (BMI), waist circumference, hip circumference, and waist-to-height ratio. BMI was calculated as body weight in kilograms divided by the square of height in meters. Body composition was assessed using the Fresenius Body Composition Monitor (BCM; Fresenius Medical Care Deutschland GmbH, Bad Homburg, Germany), a multifrequency bioelectrical impedance spectroscopy device that applies 50 discrete frequencies between 5 kHz and 1000 kHz and derives fat mass, lean tissue mass, and fluid compartments from a validated three-compartment physiological model. A single device unit was used for all baseline and 12-month assessments across the study period (June 2024 to December 2025); the manufacturer’s internal calibration check was performed before each measurement session, and disposable pre-gelled electrodes were applied to the dominant hand and ipsilateral foot with patients examined in the supine position after at least 5 min of rest, in accordance with standard device procedures. Percentage fat mass, absolute fat mass, lean mass, and skeletal muscle mass were recorded. Bioelectrical impedance analysis has been described as a feasible method for estimating body-composition compartments in clinical settings and has been specifically evaluated in relation to sarcopenia and T2DM [24,25,26].

Muscle strength was assessed using a Saehan hydraulic hand dynamometer (model SH5001, Saehan Corporation, Changwon, Republic of Korea), with a measurement range of 0–90 kg and 2 kg graduation. The same dynamometer unit was used throughout the study and its calibration was verified against the manufacturer’s reference weights before the start of the study period and at 6-month intervals thereafter. Measurements were made on the dominant hand with the participant seated, shoulder adducted and neutrally rotated, elbow flexed at 90 degrees, and forearm and wrist in neutral position; the handle was set at the second position. Three maximal voluntary contractions were performed with 60 s of rest between attempts, and the best of the three was used for analysis. Because handgrip strength is sensitive to the measurement protocol, including hand tested, posture, arm position, and dynamometer setting, this standardized approach was applied uniformly at baseline and at 12 months, by the same trained operator [38]. In the present study, handgrip strength was treated primarily as a measure of functional preservation rather than a measure of weight loss, consistent with its role as a clinically interpretable marker of muscle function [27].

Glycemic and biochemical assessments included HbA1c, fasting blood glucose, insulin, C-peptide, HOMA-IR, lipid profile, serum creatinine, estimated glomerular filtration rate, urea, urinary albumin, and albumin-to-creatinine ratio. HOMA-IR was used as a fasting surrogate index of insulin resistance, derived from fasting glucose and insulin concentrations [39]. HbA1c was measured by high-performance liquid chromatography. Other biochemical variables were measured using standardized automated methods in the institution’s certified laboratory. Inflammatory markers included erythrocyte sedimentation rate, C-reactive protein, high-sensitivity C-reactive protein, fibrinogen, and white blood cell count; inflammatory outcomes were considered secondary in the present analysis.

All patients received standard-of-care dietary and lifestyle counseling as part of routine outpatient diabetes management at the study center. This counseling followed a uniform institutional protocol, was delivered by the same clinical team, addressed energy balance, carbohydrate distribution, adequate protein intake, and regular physical activity, and was not tailored to the antidiabetic regimen. Habitual physical activity, structured resistance exercise, and dietary protein intake were not quantified using accelerometry, validated activity questionnaires, or dietary recall instruments, and could therefore not be entered as covariates in the adjusted models. Because counseling was protocol-uniform and treatment allocation was driven by glycemic control, cardiorenal indication, and reimbursement eligibility rather than by a patient’s lifestyle profile, systematically differential lifestyle intervention between treatment groups is unlikely. Unmeasured between-patient variation in physical activity and protein intake nevertheless remains a plausible contributor to the observed lean-mass and handgrip outcomes and cannot be separated from treatment-associated effects within this design; this is addressed explicitly in Section 4.6.

### 2.5. Definition of Change Variables and Derived Indices

For each patient, the absolute change from baseline at 12 months was calculated as the 12-month value minus the baseline value. Therefore, negative values for body weight, fat mass, fat mass percentage, lean mass, skeletal muscle mass and HbA1c indicated reductions over time.

Relative loss percentages were calculated as the difference between baseline and 12-month values, divided by baseline and multiplied by 100. Using this convention, positive values for weight loss, fat-mass loss, lean-mass loss, and skeletal-muscle-mass loss indicated a reduction from baseline.

The main body-composition variables analyzed longitudinally were absolute and percentage changes in body weight, absolute fat mass, percentage fat mass, lean mass, and skeletal muscle mass. Functional change was assessed as absolute change in handgrip strength from baseline to 12 months.

Additional derived indices were calculated to capture the qualitative relationship between adiposity and functional reserve. These included the fat-to-lean mass ratio, handgrip strength divided by body weight, and handgrip strength divided by skeletal muscle mass. The fat-to-lean mass ratio was used as an exploratory index of body-composition balance, consistent with evidence that relationships between lean mass and fat or fat-to-muscle can provide clinically relevant information about cardiometabolic risk beyond isolated body-composition compartments [40]. The ratios of handgrip mass to weight and skeletal muscle mass were used as exploratory indices of relative functional capacity, supported by studies showing that relative handgrip strength may be more informative than absolute handgrip strength for assessing metabolic health [41].

### 2.6. Primary Myoprotective Fat-Loss Phenotype

The primary construct of the present analysis was the myoprotective fat-loss phenotype. This phenotype was designed to distinguish simple weight loss from functionally favorable adiposity reduction, consistent with the concept that high-quality weight loss should preferentially reduce adiposity while preserving lean mass, skeletal muscle, and physical function [20,21,22,23]. A patient was classified as having a myoprotective fat-loss phenotype if all of the following criteria were met:

1. Relative reduction in absolute fat mass of at least 5% from baseline to 12 months;
2. Preservation of lean mass, defined as relative lean-mass loss < 3%;
3. Preservation of handgrip strength, defined as no decline greater than 1 kg from baseline to 12 months.

The 5% fat-mass reduction threshold was selected as a clinically interpretable adiposity response criterion, conceptually aligned with the established use of ≥5% weight loss as the threshold for clinically meaningful metabolic benefit in obesity-associated T2DM [8]. Because the present analysis focused specifically on body composition rather than total body weight, the threshold was applied to absolute fat mass rather than body weight. Preservation of lean mass and preservation of handgrip were treated as functional quality criteria, consistent with the clinical relevance of muscle preservation and handgrip strength as markers of muscle function [21,22,23,27,38].

Preservation of lean muscle mass was defined using an exploratory threshold of relative loss of <3%. This threshold was used to identify patients in whom fat-mass reduction occurred without a concomitant relevant loss of lean muscle mass, consistent with the broader concept that high-quality weight loss should preserve as much muscle and muscle compartments as possible [20,21,22,23]. Preservation of grip strength was defined as no loss greater than 1 kg, as the clinical question was not whether strength improved during weight loss, but whether weight loss was accompanied by measurable deterioration in muscle function. Handgrip strength was therefore prioritized as a marker of functional preservation, consistent with its clinical relevance in assessing muscle function [27,38].

Skeletal-muscle-mass preservation, defined as a relative loss of skeletal muscle mass <3%, was analyzed as a complementary secondary endpoint but was not included in the primary myoprotective phenotype. This decision was made to maintain a clinically interpretable primary phenotype and to prioritize handgrip strength as a functional endpoint.

### 2.7. Mutually Exclusive Weight-Loss Quality Phenotypes

Patients were further classified into four mutually exclusive weight-loss quality phenotypes:

1. Myoprotective fat-loss phenotype: fat-mass reduction ≥ 5%, lean-mass loss < 3%, and handgrip decline ≤ 1 kg.
2. Mixed fat-loss with muscle/function cost: fat-mass reduction ≥ 5%, but lean-mass loss ≥ 3% and/or handgrip decline > 1 kg.
3. Low fat-loss but functionally stable phenotype: fat-mass reduction <5%, no fat-mass gain, and handgrip decline ≤ 1 kg.
4. Non-fat-loss/functional-risk phenotype: fat-mass reduction < 5% together with either fat-mass gain or handgrip decline > 1 kg.

These categories were intended to separate patients with favorable adiposity reduction and preserved function from those with inadequate fat mass response or possible functional vulnerability, consistent with the broader concept that the quality of weight loss should consider adiposity reduction along with preservation of lean mass and muscle function [20,21,22,23,27]. HbA1c reduction was not included in the definition of the primary phenotype, as the purpose of the present analysis was to assess the functional quality of weight loss rather than to replicate the phenotyping of glycemic response. HbA1c was retained as a secondary metabolic outcome.

### 2.8. Study Outcomes

The primary outcome was the proportion of patients achieving the myoprotective fat-loss phenotype in the overall cohort and within each treatment group.

Secondary outcomes included:

1. Distribution of the four mutually exclusive weight-loss quality phenotypes by treatment group;
2. Frequency of each individual response criterion: fat-mass reduction ≥ 5%, lean-mass preservation, skeletal-muscle-mass preservation, and handgrip preservation;
3. Unadjusted 12-month changes in body weight, fat mass, fat mass percentage, lean mass, skeletal muscle mass, handgrip strength, fat-to-lean ratio, handgrip-to-weight ratio, handgrip-to-skeletal-muscle-mass ratio, and HbA1c by treatment group;
4. Baseline clinical and metabolic characteristics according to myoprotective phenotype status;
5. Adjusted 12-month body-composition and functional outcomes by treatment group;
6. Correlations between fat-mass loss, lean-mass loss, and handgrip changes.

### 2.9. Statistical Analysis

All statistical analyses were performed in Python (v3.12; Python Software Foundation, Wilmington, DE, USA; using the statsmodels, SciPy, NumPy, and pandas packages.

The primary analysis included the 166 patients who completed the 12-month follow-up period without treatment change and who had the variables required for classification of the myoprotective phenotype. Missing values were not imputed; analyses were performed using complete cases for each specified outcome.

Continuous variables were summarized as mean ± standard deviation and, where applicable, median with interquartile range. Categorical variables were summarized as counts and percentages. Unadjusted changes from baseline to 12 months were summarized by treatment group. Global between-group comparisons of continuous change variables were performed using Kruskal–Wallis tests due to unequal treatment group sizes and potential non-normality of change distributions [42]. Categorical comparisons were performed using chi-square tests, with sparse cells interpreted with caution.

The association between treatment group and the mutually exclusive weight loss quality phenotype was assessed using a χ^2^ statistic. Because several phenotype-by-treatment cells were sparse, permutation testing was used to support the interpretation of the global phenotype-treatment association and to reduce reliance on asymptotic assumptions.

Adjusted continuous outcomes were analyzed using ANCOVA regression models with HC3 robust standard errors consistent with heteroskedasticity. The dependent variable was the outcome value at 12 months. Metformin monotherapy was used as the control group. Each model included the treatment group and the baseline value of the corresponding outcome, with additional adjustments for age, sex, duration of diabetes, and baseline BMI. This baseline-adjusted modeling strategy was selected to improve precision and account for baseline differences in the corresponding outcome [43]. HC3 robust standard errors were used to reduce the sensitivity to heteroskedasticity in the regression models [44]. Analyses were performed adjusting for weight, fat mass, percent fat mass, lean mass, skeletal muscle mass, handgrip strength, fat-to-lean mass ratio, handgrip-to-weight ratio, handgrip-to-skeletal-muscle-mass ratio, and HbA1c.

Spearman correlation analyses were used to assess associations between adiposity reduction and muscle/functional changes, including percent fat-mass loss, percent lean-mass loss, change in handgrip strength, change in fat-to-lean mass ratio, change in handgrip-to-weight ratio, and change in handgrip-to-skeletal-muscle-mass ratio.

Multiplicity was addressed using the Benjamini–Hochberg correction for false discovery rate [45]. FDR-adjusted q-values were reported for unadjusted continuous comparisons between groups, binary response criteria, adjusted treatment contrasts, and correlation analyses. Statistical tests were two-sided. Nominal *p*-values < 0.05 were reported, while FDR-adjusted q-values were used to guide interpretation in families of related analyses.

No a priori sample-size or power calculation was performed, either for the overall cohort or for the treatment subgroups. The cohort size was determined by the number of eligible patients with complete paired baseline and 12-month assessments available at the study center during the recruitment window, rather than by a target precision. Post hoc, the smaller subgroups afford limited power: for a two-sided one-sample comparison at alpha = 0.05, 80% power corresponds to a standardized effect of approximately 1.00 SD at *n* = 10 (sulfonylurea group), 0.72 SD at *n* = 17 (DPP-4 inhibitor group), and 0.61 SD at *n* = 23 (GLP-1 receptor agonist group). Only large effects are therefore detectable in the sulfonylurea and DPP-4 inhibitor strata, and findings in these two groups are reported as descriptive and hypothesis-generating. In particular, the observed myoprotective phenotype rate of 0/10 in the sulfonylurea group is compatible with a true rate as high as 30.8% (95% Clopper–Pearson interval 0.0–30.8%), and should be interpreted as consistent with a low rate rather than as evidence of a true zero. By contrast, the corresponding rate of 0/45 in the insulin group carries an upper 95% bound of 7.9%, which does support a genuinely low phenotype frequency in that group. Confidence intervals for subgroup proportions are reported throughout to make this asymmetry in precision explicit.

## 3. Results

### 3.1. Analytic Cohort and Overall Attainment of Myoprotective Criteria

The final analytic cohort included 166 adults with T2DM who completed 12-month follow-up without treatment change and had the variables required for body-composition and functional-response classification. The treatment groups were metformin monotherapy (*n* = 46), metformin plus sulfonylurea (SU; *n* = 10), metformin plus dipeptidyl peptidase-4 inhibitor (DPP-4i; *n* = 17), metformin plus sodium-glucose cotransporter-2 inhibitor (SGLT2i; *n* = 25), metformin plus glucagon-like peptide-1 receptor agonist (GLP-1 RA; *n* = 23), and metformin plus insulin (*n* = 45).

**Figure 1:**
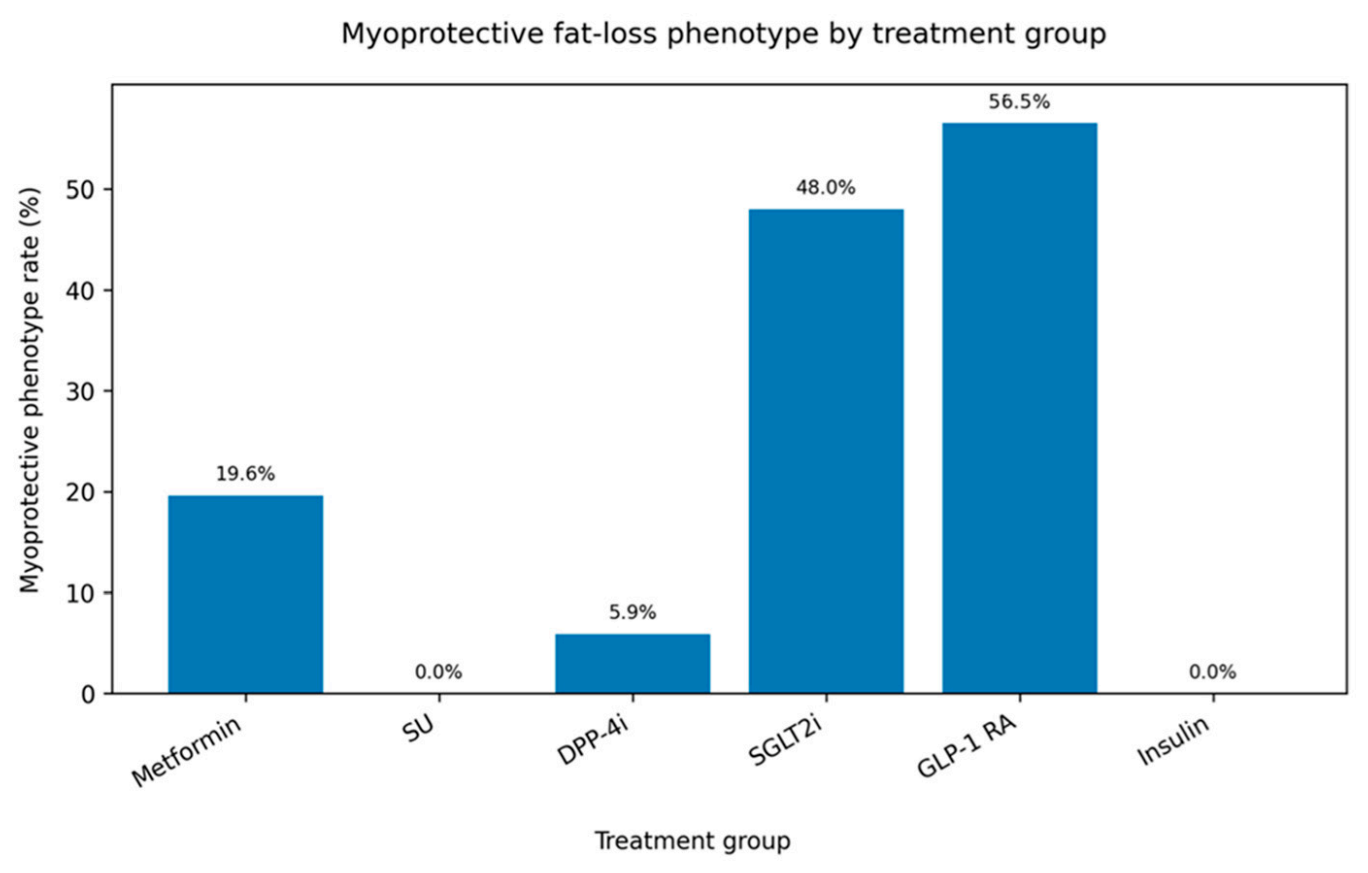
Myoprotective fat-loss phenotype rate by treatment group. The phenotype was defined as fat-mass reduction ≥ 5%, lean-mass loss < 3%, and handgrip decline ≤ 1 kg. All six groups received metformin as background therapy; groups are labeled “Metformin” for monotherapy and “Metformin + [agent]” for each combination regimen, and none of the combination groups represents monotherapy with the added agent.

Overall, 58 of 166 patients (34.9%) achieved a fat-mass reduction of at least 5%. Lean-mass preservation, defined as relative lean-mass loss < 3%, was observed in 129 patients (77.7%). Skeletal-muscle-mass preservation, defined as relative skeletal-muscle-mass loss < 3%, was observed in 155 patients (93.4%), and handgrip-strength preservation, defined as no decline greater than 1 kg, was observed in 143 patients (86.1%). The full myoprotective fat-loss phenotype, defined as simultaneous fat-mass reduction ≥ 5%, lean-mass preservation, and handgrip preservation, was present in 35 patients (21.1%). The myoprotective phenotype rate by treatment group is shown in Figure 1, and individual response-criterion achievement is summarized in Table 1.

### 3.2. Distribution of Mutually Exclusive Weight-Loss Quality Phenotypes

The distribution of mutually exclusive weight-loss quality phenotypes differed markedly across treatment groups (χ^2^ = 131.58; permutation *p* < 0.0001). In the overall cohort, 35 patients (21.1%) had a myoprotective fat-loss phenotype, 23 (13.9%) had mixed fat loss with muscle/function cost, 42 (25.3%) had low fat loss with functional stability, and 66 (39.8%) had a non-fat-loss/functional-risk phenotype.

**Figure 2:**
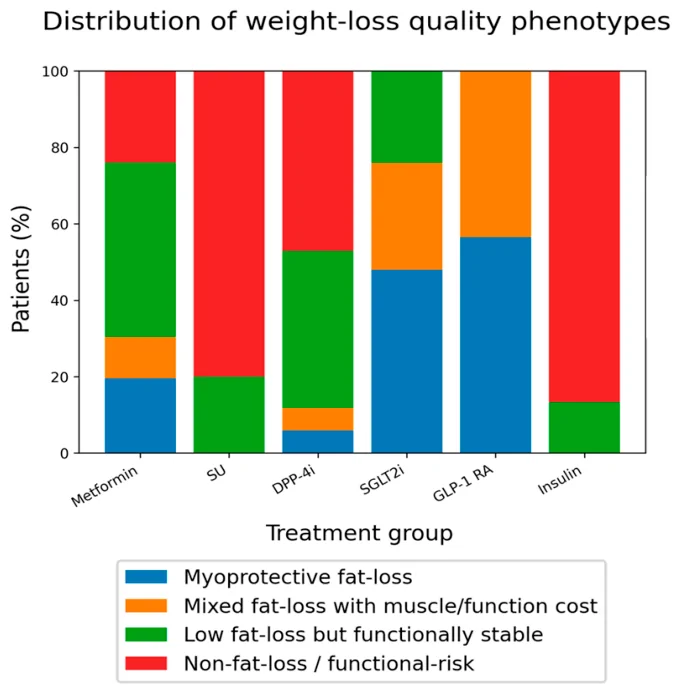
The distribution of mutually exclusive weight-loss quality phenotypes by treatment group. Percentages are calculated within each treatment group. All six groups received metformin as background therapy; groups are labeled “Metformin” for monotherapy and “Metformin + [agent]” for each combination regimen, and none of the combination groups represents monotherapy with the added agent.

The myoprotective phenotype was most frequent in the metformin plus GLP-1 RA group (13/23; 56.5%) and the metformin plus SGLT2i group (12/25; 48.0%). It was less frequent with metformin monotherapy (9/46; 19.6%) and metformin plus DPP-4i (1/17; 5.9%) and was not observed in the sulfonylurea (0/10) or insulin (0/45) groups. These two zero counts differ materially in precision and should not be read as equivalent: the 95% Clopper–Pearson interval for 0/45 in the insulin group is 0.0–7.9%, which supports a genuinely low phenotype frequency, whereas the interval for 0/10 in the sulfonylurea group extends to 30.8% and does not exclude a rate comparable to that observed with metformin monotherapy. The sulfonylurea result is therefore reported as consistent with a low rate rather than as an established absence. Conversely, the non-fat-loss/functional-risk phenotype predominated in the insulin group (39/45; 86.7%) and the sulfonylurea group (8/10; 80.0%). The full distribution of mutually exclusive weight-loss quality phenotypes is illustrated in Figure 2 and summarized in Table 2.

### 3.3. Unadjusted 12-Month Body-Composition, Functional, and Glycemic Changes

Unadjusted 12-month changes showed substantial treatment-group differences in weight and adiposity-related outcomes. Mean percentage weight loss was greatest in the GLP-1 RA group (6.48 ± 1.56%) and the SGLT2i group (3.22 ± 1.25%). By contrast, negative percentage weight-loss values were observed in the insulin group (−1.92 ± 1.44%) and the sulfonylurea group (−1.65 ± 1.20%), indicating mean weight gain over 12 months. Between-group differences were significant for both absolute weight change and percentage weight loss (both FDR q < 0.0001).

Fat-mass loss showed the clearest separation across treatment groups. Mean relative fat-mass loss was 14.76 ± 4.33% in the GLP-1 RA group and 7.13 ± 2.69% in the SGLT2i group, compared with 2.97 ± 3.43% with metformin monotherapy and 1.21 ± 3.70% with DPP-4i. In contrast, mean fat mass increased in the sulfonylurea and insulin groups, as reflected by negative fat-mass loss percentages (−2.04 ± 3.60% and −3.36 ± 3.48%, respectively). The between-group difference in relative fat-mass loss was highly significant (Kruskal–Wallis *p* < 0.0001; FDR q < 0.0001).

**Figure 3:**
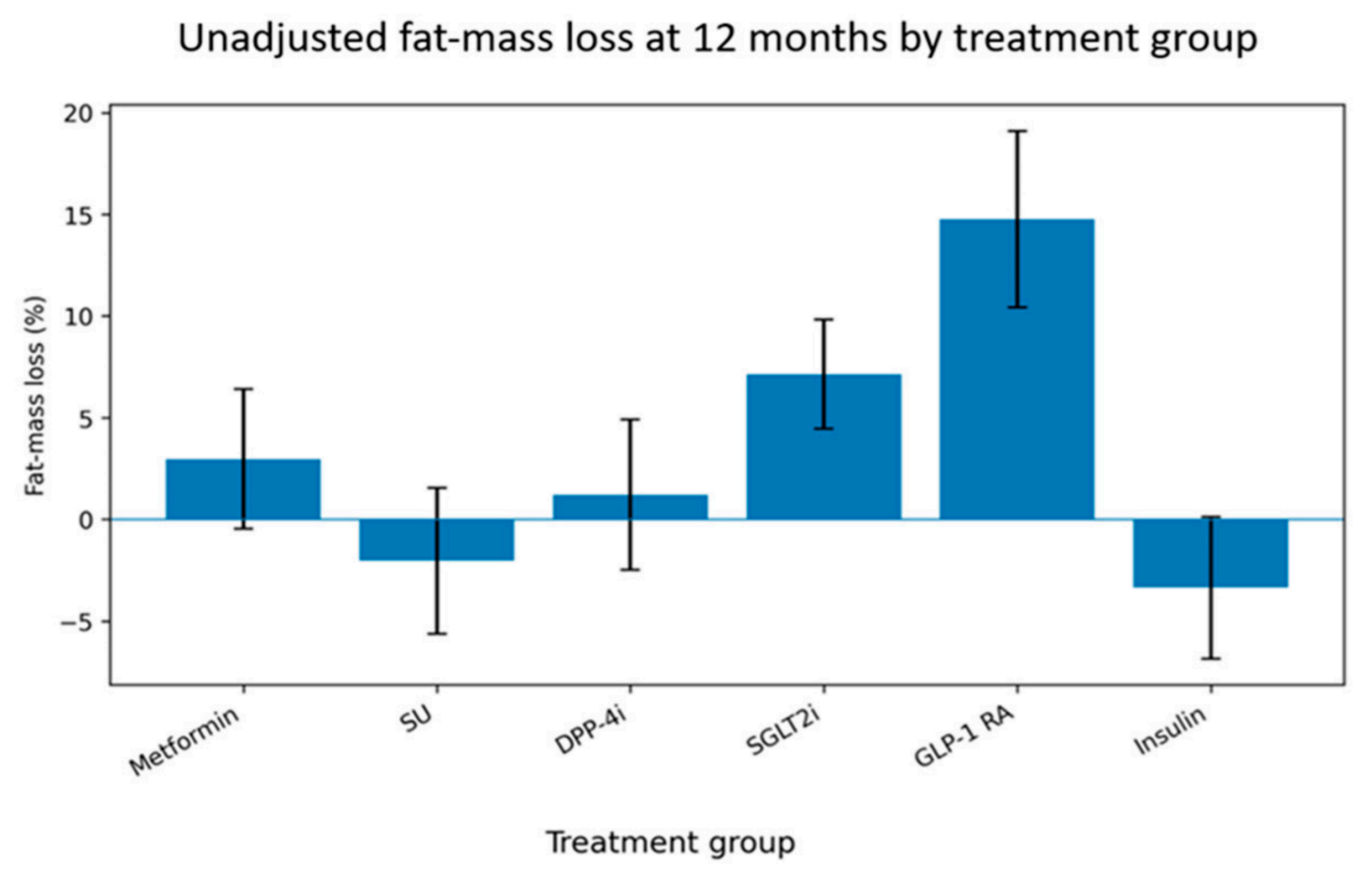
Unadjusted relative fat-mass loss at 12 months by treatment group. The bars represent mean values and the error bars represent standard deviations. Positive values indicate reduction from baseline. All six groups received metformin as background therapy; groups are labeled “Metformin” for monotherapy and “Metformin + [agent]” for each combination regimen, and none of the combination groups represents monotherapy with the added agent.

In contrast to adiposity-related outcomes, unadjusted changes in lean mass, skeletal muscle mass, and handgrip strength did not differ significantly across treatment groups after FDR correction. Mean handgrip strength increased modestly in all treatment groups, with no significant global between-group difference (*p* = 0.797; FDR q = 0.797). HbA1c decreased in all treatment groups, with the largest unadjusted reductions observed in the GLP-1 RA and insulin groups. Unadjusted relative fat-mass loss is illustrated in Figure 3, and unadjusted 12-month changes are summarized in Table 3.

### 3.4. Baseline Characteristics According to Weight-Loss Quality Phenotype

Baseline clinical and metabolic characteristics were broadly similar across the four weight-loss quality phenotypes. No baseline variable differed significantly across phenotypes after FDR correction. The myoprotective fat-loss group had a mean age of 56.4 ± 13.1 years, included 62.9% women, and had a mean diabetes duration of 14.0 ± 8.3 years, baseline BMI of 36.5 ± 4.9 kg/m^2^, baseline fat mass of 41.7 ± 9.8 kg, and baseline handgrip strength of 30.4 ± 11.4 kg.

Baseline BMI showed the lowest unadjusted *p*-value across phenotypes (*p* = 0.056), with lower mean BMI in the mixed fat-loss with muscle/function cost group; however, this difference was not significant after multiplicity correction (FDR q = 0.673). Baseline HbA1c, fat mass percentage, absolute fat mass, lean mass, skeletal muscle mass, handgrip strength, and hs-CRP showed no statistically robust differences across phenotypes. Baseline characteristics according to weight-loss quality phenotype are summarized in Table 4.

### 3.5. Adjusted Treatment Contrasts for Body-Composition and Functional Outcomes

In adjusted ANCOVA-type models using metformin monotherapy as the reference group, treatment-group differences in adiposity-related outcomes remained evident after adjustment for the baseline value of the corresponding outcome, age, sex, diabetes duration, and baseline BMI. Compared with metformin monotherapy, the metformin plus GLP-1 RA group had lower 12-month fat mass (β = −4.689 kg, 95% CI −5.462 to −3.916; FDR q < 0.0001), lower fat mass percentage (β = −2.731 percentage points, 95% CI −3.266 to −2.195; FDR q < 0.0001), and a lower fat-to-lean ratio (β = −0.074, 95% CI −0.095 to −0.054; FDR q < 0.0001).

The metformin plus SGLT2i group showed a similar but smaller adjusted adiposity profile, with lower 12-month fat mass (β = −1.650 kg, 95% CI −2.240 to −1.060; FDR q < 0.0001), lower fat mass percentage (β = −0.901 percentage points, 95% CI −1.317 to −0.484; FDR q < 0.0001), and lower fat-to-lean ratio (β = −0.028, 95% CI −0.045 to −0.011; FDR q = 0.003) compared with metformin monotherapy. By contrast, the metformin plus insulin group had higher adjusted fat mass, fat mass percentage, and fat-to-lean ratio compared with metformin monotherapy.

**Figure 4:**
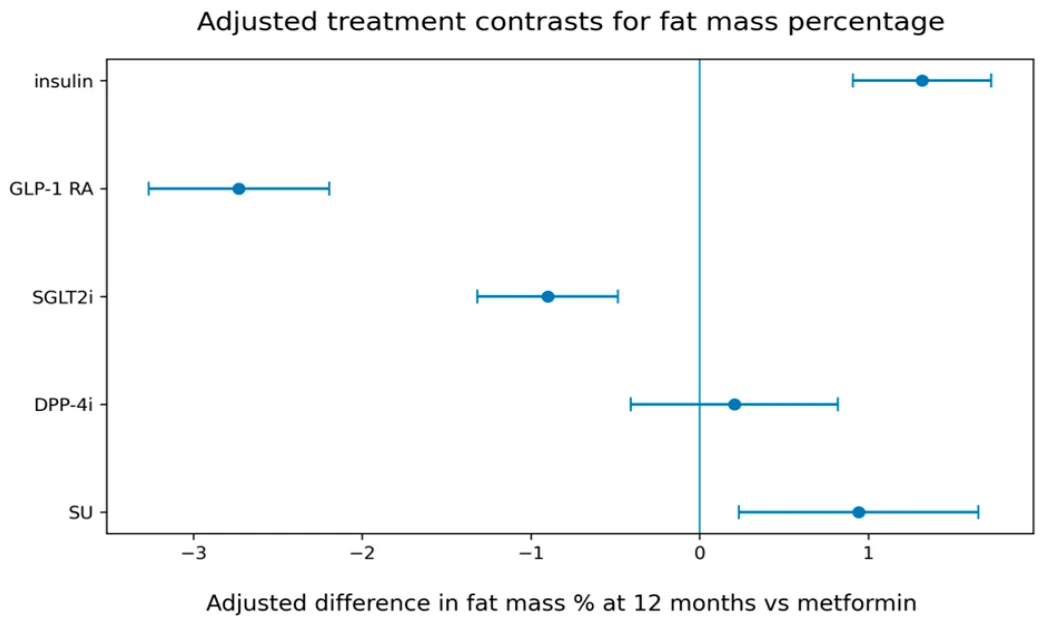
The adjusted treatment contrasts for fat mass percentage at 12 months. The points represent adjusted differences versus metformin monotherapy; the horizontal bars represent 95% confidence intervals. All comparator groups received metformin as background therapy and are labeled “Metformin + [agent]”; none represents monotherapy with the added agent.

Adjusted models did not show significant treatment-group differences in 12-month lean mass or handgrip strength after FDR correction. The metformin plus GLP-1 RA group showed a higher handgrip-to-weight ratio (β = 2.081 units on the ×100 scale, 95% CI 1.065 to 3.098; FDR q = 0.0002), whereas the metformin plus insulin group showed a lower handgrip-to-weight ratio (β = −0.992 units on the ×100 scale, 95% CI −1.686 to −0.298; FDR q = 0.014). Adjusted treatment contrasts for fat mass percentage are shown in Figure 4, and selected adjusted treatment contrasts are summarized in Table 5.

### 3.6. Patient-Level Relationships Between Adiposity Loss and Muscle/Function Changes

At the patient level, relative weight loss was strongly correlated with relative fat-mass loss (Spearman ρ = 0.948; FDR q < 0.0001), suggesting that body-weight reduction largely tracked adiposity reduction in this cohort. Weight loss was also modestly correlated with lean-mass loss (ρ = 0.286; FDR q = 0.0004), and fat-mass loss was weakly correlated with lean-mass loss (ρ = 0.174; FDR q = 0.044).

**Figure 5:**
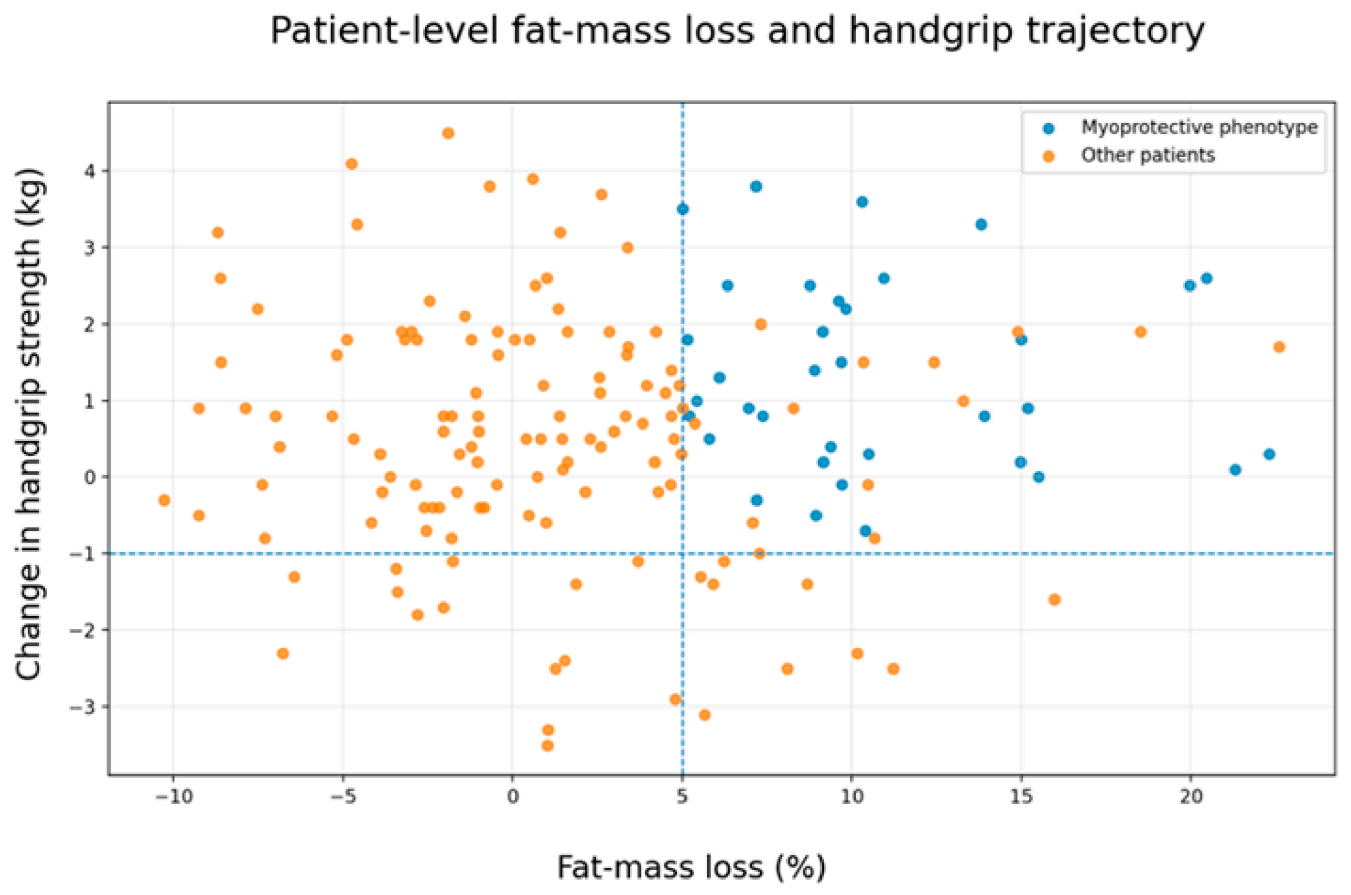
Patient-level fat-mass loss and handgrip change. The vertical dashed line indicates the 5% fat-mass reduction threshold; the horizontal dashed line indicates a 1 kg decline in handgrip strength. Points meeting both thresholds and the lean-mass preservation criterion were classified as the myoprotective phenotype.

Relative fat-mass loss was not significantly correlated with change in handgrip strength (ρ = 0.060; FDR q = 0.518) or with change in handgrip-to-skeletal-muscle-mass ratio (ρ = 0.049; FDR q = 0.534). However, fat-mass loss was positively correlated with change in handgrip-to-weight ratio (ρ = 0.496; FDR q < 0.0001), consistent with improved relative functional capacity when adiposity decreased without parallel functional deterioration. The patient-level relationship between fat-mass loss and handgrip change is illustrated in Figure 5, and correlation analyses are summarized in Table 6.

Taken together, these results indicate that clinically meaningful fat-mass reduction was concentrated in the GLP-1 RA and SGLT2i groups and was not accompanied by detectable group-level deterioration in lean mass or handgrip strength. The myoprotective fat-loss phenotype captured a distinct response pattern: adiposity reduction with preservation of muscle-related functional reserve.

## 4. Discussion

### 4.1. Principal Findings

In this 12-month, real-world cohort of adults with obesity-related T2DM, the present analysis shifted the focus from the magnitude of weight loss to its functional quality, consistent with the emerging concept that favorable weight loss should preferentially reduce adiposity while preserving lean mass and muscle function [20,21,22,23]. The main finding was that only a subset of patients achieved a myoprotective fat-loss phenotype, defined as a prespecified reduction in fat mass accompanied by preservation of lean mass and handgrip strength. Although 58 of 166 patients (34.9%) achieved at least a 5% reduction in fat mass, only 35 patients (21.1%) achieved the full myoprotective phenotype. This distinction is clinically relevant because adiposity reduction, lean-mass preservation, and functional preservation do not necessarily converge in all patients, supporting a patient-level phenotypic approach to therapeutic response [28,29,30,31].

The distribution of this phenotype varied substantially between treatment groups. Myoprotective fat loss was most common in the metformin plus GLP-1 RA and metformin plus SGLT2i groups, occurring in 56.5% and 48.0% of patients, respectively. In contrast, the phenotype was not observed in the insulin group, in which the sample size supports a genuinely low frequency, nor in the sulfonylurea group, where the small stratum (*n* = 10) makes the observed zero rate compatible with a substantially higher true rate; it was also less common with DPP-4 inhibitors. Unadjusted results showed the clearest separation for fat loss, with the GLP-1 RA group showing the greatest relative mean reduction in fat mass, followed by the SGLT2i group. This pattern is generally consistent with the literature showing favorable effects of GLP-1 RA and SGLT2 inhibitor therapies on body weight and adiposity-related outcomes, although body-composition responses vary across interventions, populations, and assessment methods [10,13,14]. In contrast, changes in lean mass, skeletal muscle mass, and handgrip strength did not differ significantly between treatment groups after correction for multiple testing. Because treatment was not randomized and regimen was used as a descriptive stratification factor rather than as a comparator arm (Section 2.3), these differences are reported as baseline-adjusted descriptive contrasts between non-randomized strata and not as comparative treatment effects.

The adjusted models supported the same pattern. Compared with metformin monotherapy, the metformin plus GLP-1 RA group had lower 12-month adjusted fat mass, lower percent fat mass, lower fat-to-lean mass ratio, and higher handgrip-to-weight ratio. The metformin plus SGLT2i group had a similar but more moderate adiposity profile. The metformin plus insulin group had the opposite pattern, with higher adjusted fat mass, higher percent fat mass, higher fat-to-lean mass ratio, and lower handgrip-to-weight ratio, compared with metformin monotherapy. Because handgrip strength normalized to body weight may capture relative functional capacity more directly than absolute handgrip strength alone, these findings suggest that reducing adiposity may improve functional reserve relative to body size when muscle function is preserved [27,41]. At the patient level, loss of fat mass was not significantly correlated with change in handgrip strength, suggesting that reducing adiposity was not accompanied by measurable functional impairment in this cohort.

### 4.2. Functional Quality of Weight Loss as a Clinically Relevant Construct

Current diabetes care increasingly recognizes weight management as a central component of T2DM treatment, rather than a secondary cosmetic goal [46]. However, total body weight is a composite measure that cannot distinguish fat loss from lean tissue loss, fluid changes, or changes in muscle-related functional reserve. This limitation is particularly relevant in patients with long-standing T2DM, obesity, age-related frailty, or risk of sarcopenic obesity, in whom weight reduction may be metabolically beneficial but potentially undesirable if accompanied by a relevant decrease in muscle mass or strength [47,48].

The present analysis operationalized this issue by defining a phenotype that required both adiposity reduction and functional protection. The use of handgrip strength as a central component is consistent with evidence supporting grip strength as a clinically informative biomarker of muscle function, functional status, multimorbidity, and adverse health outcomes in older adults and metabolically vulnerable populations [49]. Similarly, the use of bioimpedance-derived fat and lean mass compartments is aligned with the practical role of body-composition assessment in clinical and metabolic research, while recognizing that BIA remains method-dependent and sensitive to hydration status, body position, and population-specific assumptions [50].

This construct complements, rather than replaces, conventional glycemic and weight-centered endpoints. HbA1c reduction remains essential in T2DM, but a glucose-centered response does not describe whether treatment improves the adiposity-driven component of the disease or whether weight loss occurs with preservation of functional reserve. Therefore, the myoprotective phenotype may provide a clinically interpretable bridge between metabolic efficacy, body-composition remodeling, and functional safety.

### 4.3. Interpretation of Treatment-Associated Patterns

The metformin plus GLP-1 receptor agonist group demonstrated the strongest response to adiposity, with all patients achieving at least a 5% reduction in fat mass and more than half achieving the full myoprotective phenotype. This finding is consistent with recent reviews and clinical evidence showing that GLP-1 receptor agonist-based therapies reduce body weight largely by reducing fat mass, although reductions in lean mass may also occur, which appear to vary depending on the molecule, population, duration of treatment, and method of body-composition assessment [51,52,53]. In the present cohort, the GLP-1 RA group showed no detectable adjusted disadvantage in handgrip strength, while the handgrip strength-to-weight ratio was greater than in the metformin monotherapy group. This finding suggests improved relative functional capacity in parallel with reduced adiposity and is consistent with evidence that relative handgrip strength may be more informative than absolute handgrip strength alone for assessing cardiometabolic risk [54].

One consequence of applying a fixed 5% fat-mass threshold deserves comment. In the GLP-1 RA group all 23 patients exceeded this threshold, as did 19 of 25 patients in the SGLT2i group, and both groups exceeded it by a wide margin (mean relative fat-mass loss 14.76% and 7.13%, respectively). Within these two strata the adiposity criterion is therefore close to saturated and carries little discriminating information, so membership of the myoprotective phenotype is determined almost entirely by the lean-mass and handgrip criteria. This is a property of a dichotomous threshold applied to groups with a strong adiposity response, and it means that the phenotype rate should not be read as a measure of the magnitude of fat loss in these groups. The relationship between the magnitude of adiposity reduction and muscle or functional outcomes is instead addressed on a continuous scale in Section 3.6 and Table 6, where relative fat-mass loss was only weakly correlated with lean-mass loss and showed no significant association with change in handgrip strength. Because second-line agents were prescribed at fixed maintenance doses with no titration during follow-up (Section 2.3), a dose–response analysis within these groups was not estimable in this cohort.

At the same time, the results of the GLP-1 receptor agonist trial should not be interpreted as evidence of universal muscle protection. A substantial proportion of patients in this group were classified as having mixed fat loss with muscle/functional cost, because fat loss occurred either in conjunction with loss of lean mass above a predefined exploratory threshold or with reduced handgrip capacity. This nuance is consistent with the recent literature emphasizing that lean-mass and muscle-related responses to GLP-1-based therapies are heterogeneous and may be adaptive in some patients but potentially unfavorable in others [55,56]. At the group level, mean handgrip strength was maintained; at the individual level, however, some patients may still require monitoring of lean mass, muscle strength, dietary protein intake, and resistance training during pharmacologically induced weight loss [57,58].

The SGLT2 inhibitor group showed a smaller but clinically consistent pattern: moderate fat-mass loss, a high rate of the myoprotective phenotype, and no detectable impairment in handgrip strength. This pattern is consistent with recent meta-analytic evidence suggesting that long-term SGLT2 inhibitor therapy reduces body weight and fat mass in patients with T2DM, while effects on fat-free mass and muscle compartments appear smaller and remain clinically relevant at follow-up [59]. Recent real-world data also support the concept that weight loss associated with SGLT2 inhibitors may be predominantly attributable to fat mass, rather than fat-free mass, and may occur without relevant impairment in muscle strength [60]. The current findings add to this literature by applying an integrated phenotype rather than examining isolated body-composition compartments.

Neither the insulin nor the sulfonylurea group contained a patient meeting the full myoprotective definition, and both showed a predominance of phenotypes without fat loss or with functional risk. This finding should be interpreted with caution due to confounding by indication and the observational design. Patients receiving insulin may represent a more advanced or metabolically complex subgroup, and insulin exposure was analyzed as a heterogeneous category, without stratification by basal, premixed, or basal-bolus regimen or by dose. However, the observed association between insulin group status and higher adjusted fat mass is biologically plausible and consistent with the recognized tendency of insulin therapy to promote weight gain in T2DM [61]. The pattern observed in the sulfonylurea group is directionally consistent with evidence that insulin secretagogues generally promote weight gain [62], but with only 10 patients this stratum cannot support a claim about the frequency of myoprotective response and is reported as hypothesis-generating. These data support the need to assess body composition and functional reserve when glycemic-lowering strategies are associated with weight gain.

### 4.4. Body Composition, Sarcopenic-Obesity Risk, and Patient-Level Heterogeneity

A central implication of this analysis is that, with regard to body composition, information is not captured by BMI or weight alone. In older adults with T2DM, a higher percentage of body fat has been associated with an increased risk of sarcopenia, while BMI may mask adverse fat–muscle relationships because it does not differentiate adiposity from lean tissue [63]. This limitation is particularly relevant in sarcopenic obesity, where low muscle mass and high fat mass may coexist despite apparently non-extreme or even normal body dimensions, with adverse metabolic implications [64]. In the present cohort, weight loss was strongly correlated with fat-mass loss, indicating that weight reduction largely reflected a reduction in adiposity. However, weight loss was also modestly correlated with lean-mass loss, reinforcing the need to monitor the composition of weight changes rather than assuming that all weight reductions are functionally favorable.

The absence of significant differences at baseline between weight loss quality phenotypes after adjustment for false discovery rate suggests that the myoprotective response was not easily predicted by simple baseline characteristics such as age, sex, BMI, baseline fat mass, handgrip strength, HbA1c, or hs-CRP. This supports the broader concept of heterogeneity in obesity-associated T2DM, in which phenotype, disease progression, and response to treatment can vary substantially between patients [65]. The treatment mechanism likely contributed to the observed phenotype distribution, but unmeasured factors such as dietary intake, protein adequacy, physical activity, resistance training, adherence, dose exposure, frailty status, and disease severity may also influence individual changes.

The correlation results are particularly informative. Loss of fat mass was not significantly associated with decreased handgrip strength, whereas it was positively associated with improved handgrip strength-to-weight ratio. This suggests that in many patients, reducing adiposity may improve relative functional capacity, even if absolute strength does not change substantially. Clinically, this distinction is important: stable grip strength in the context of lower body weight and lower fat load may represent a favorable functional profile, consistent with evidence that relative grip strength may be more informative than absolute grip strength alone for interpreting metabolic risk [66].

### 4.5. Clinical Implications

The findings support a practical clinical message: in obesity-associated T2DM, the quality of weight loss, not just its magnitude, should be assessed [67,68]. A patient who loses fat mass while maintaining lean mass and handgrip strength may have a more favorable therapeutic response than a patient with similar weight loss accompanied by functional decline. Conversely, patients with inadequate fat-mass reduction or mixed fat loss and muscle/functional cost may require additional intervention, even when glycemic outcomes improve.

In routine diabetes care, this approach may encourage the inclusion of simple functional and body-composition measures in treatment evaluation. Bioimpedance-based assessment, when performed under standardized conditions, can provide useful longitudinal information on fat and lean compartments [68]. Handgrip dynamometry is inexpensive, rapid, and clinically interpretable and has been proposed as a practical biomarker of muscle function and overall health [69]. Together, these measurements may help identify patients who would benefit from nutritional counseling, resistance exercise prescription, optimization of protein intake, medication reassessment, or closer functional monitoring [67,70].

For patients treated with GLP-1 receptor agonists or SGLT2 inhibitors, the current results are reassuring at the group level, but also argue against complacency. The goal should not be just weight loss, but rather reducing adiposity while preserving functional muscle reserve. For patients treated with insulin or sulfonylureas, the results reinforce the importance of monitoring changes in weight and adiposity, especially when improvements in glycemic control occur at the cost of increased fat mass.

### 4.6. Strengths and Limitations

The main strengths of this study include the 12-month prospective design, paired assessments at baseline and follow-up, stable treatment exposure during follow-up, and the availability of body-composition and handgrip measurements in a real-world Eastern European diabetes cohort. The analysis also used clinically interpretable patient-level phenotypes rather than relying solely on mean changes in isolated variables. This approach is relevant to contemporary patient-level response phenotyping because it captures the potential discordance between adiposity response, lean-mass preservation, and functional change [30,31,65].

Several limitations should be acknowledged. First, the study was observational, and treatment allocation was not randomized; therefore, causal treatment effects cannot be inferred, and the treatment strata should be read as descriptive rather than as comparator arms. Confounding by indication is expected to be substantial: patients prescribed insulin or a sulfonylurea typically have longer diabetes duration and greater beta-cell failure, whereas GLP-1 receptor agonists and SGLT2 inhibitors are preferentially prescribed to patients with higher adiposity and cardiorenal indications, and reimbursement criteria further constrain who receives which class. Second, the treatment groups were unequal in size, with the sulfonylurea (*n* = 10) and DPP-4 inhibitor (*n* = 17) groups being particularly small, and several phenotype-by-treatment cells were sparse despite the use of global permutation-supported tests. As detailed in Section 2.9, no a priori power calculation was performed, and these two strata are powered only for large effects; the absence of the myoprotective phenotype in the sulfonylurea group is compatible with a true rate of up to 30.8% and must not be read as an established zero rate. Third, the thresholds for the myoprotective phenotype, particularly the <3% lean-mass loss criterion and the ≤1 kg handgrip decrease criterion, were clinically interpretable but exploratory; they should be validated in larger cohorts and tested against concrete functional outcomes. Fourth, restriction of the analytic cohort to patients whose regimen remained unchanged for 12 months introduces a form of selection that preferentially retains metabolically stable individuals, since patients with deteriorating control are the most likely to have had therapy intensified and thereby excluded; this is expected to bias the cohort towards more favorable body-composition trajectories and to understate the frequency of functionally adverse phenotypes in unselected clinical practice.

Fifth, body composition was assessed by bioimpedance rather than dual-energy X-ray absorptiometry, computed tomography, or magnetic resonance imaging. Although BIA is practical and suitable for routine care, estimates may be influenced by hydration status, edema, renal function, body position, and device-specific equations [50,68]. This is of particular concern in the SGLT2 inhibitor group, in which early osmotic diuresis reduces extracellular fluid and may be partly misattributed to fat or lean compartments; the 12-month assessment window mitigates but does not eliminate this, since fluid shifts largely stabilize after the first weeks of therapy. Sixth, adherence was not verified by pill count, refill records, or a validated questionnaire, and the duration of therapy before study entry was not recorded, so residual non-adherence and between-group differences in cumulative prior exposure cannot be excluded. Seventh, and most importantly for the interpretation of the muscle findings, habitual physical activity, resistance exercise, and dietary protein intake were not quantified. Skeletal muscle preservation is strongly determined by these factors, and although all patients received uniform standard-of-care lifestyle counseling and treatment allocation was not driven by lifestyle profile, the present design cannot distinguish a treatment-associated myoprotective effect from a lifestyle-associated one. The treatment-group differences reported here should therefore be understood as differences between groups of patients defined by their regimen, within which lifestyle behavior is unmeasured, rather than as pharmacological effects on muscle. Eighth, insulin regimen subtype and insulin dose were not systematically quantified; within the SGLT2 inhibitor and GLP-1 receptor agonist groups, dosing was effectively uniform at the maintenance level (Section 2.3), which removes dose heterogeneity but also precludes any dose–response analysis. Ninth, handgrip strength captures upper limb strength but does not fully characterize physical performance, gait speed, balance, lower limb function, or frailty status [27,49,69]. Finally, the study was conducted in a single tertiary center, which may limit generalizability to other populations or care settings.

### 4.7. Future Research Directions

Future studies should validate the myoprotective fat-loss phenotype in larger prospective cohorts, randomized clinical trials, and pragmatic treatment trials. Such analyses should include standardized measures of dietary intake, protein adequacy, resistance exercise, medication exposure, adherence, frailty status, walking speed, and patient-reported physical function. Imaging-based assessments, including dual-energy X-ray absorptiometry, computed tomography, or magnetic resonance imaging, would also help clarify whether myoprotective fat loss reflects favorable tissue remodeling beyond bioimpedance-detectable changes, including changes in regional adiposity, visceral fat, ectopic fat, and skeletal muscle quality [50,68].

The phenotype proposed here could also be useful as a secondary endpoint in studies of obesity-based diabetes therapy. Rather than reporting only HbA1c, body weight, or total fat mass, future studies could report the proportion of patients who achieve adiposity reduction while preserving lean mass and muscle strength. This would make treatment evaluation more aligned with the clinical goal of improving metabolic health without accelerating functional vulnerability [67,68].

## 5. Conclusions

In this real-world cohort of adults with obesity-related T2DM, clinically interpretable fat loss was not synonymous with myoprotective fat loss. The full myoprotective fat-loss phenotype occurred in approximately one-fifth of patients and was concentrated in the GLP-1 receptor agonist and SGLT2 inhibitor groups, while being absent among patients treated with insulin. The reduction in adiposity, particularly in these groups, was not accompanied by a detectable group-wide deterioration in handgrip strength, and relative functional capacity improved when expressed relative to body weight. Because treatment was not randomized, groups were unequal in size, and physical activity and protein intake were not measured; these patterns describe differences between patient strata defined by their regimen rather than pharmacological effects, and require confirmation in larger cohorts with lifestyle data.

These findings support the concept that therapeutic success in obesity-associated T2DM should be assessed by the quality of weight loss, not just glycemic control or total body weight reduction. Body-composition assessment and handgrip dynamometry may help identify patients with favorable adiposity reduction and maintained functional reserve, as well as those who require nutritional, exercise, or therapeutic optimization. Because the analysis is observational and the phenotype thresholds are exploratory, the results should be interpreted as hypothesis-generating and require validation in larger prospective studies.

## Figures and Tables

**Table 1:** Individual response-criterion achievement by treatment group.

Response Criterion	Metformin	Metformin + SU	Metformin + DPP-4i	Metformin + SGLT2i	Metformin + GLP-1 RA	Metformin + Insulin	*p*	FDR q
Fat-mass reduction ≥5%	14/46 (30.4%)	0/10 (0.0%)	2/17 (11.8%)	19/25 (76.0%)	23/23 (100.0%)	0/45 (0.0%)	<0.0001	<0.0001
Lean mass preserved (<3% loss)	34/46 (73.9%)	7/10 (70.0%)	15/17 (88.2%)	19/25 (76.0%)	14/23 (60.9%)	40/45 (88.9%)	0.114	0.191
Skeletal muscle mass preserved (<3% loss)	45/46 (97.8%)	9/10 (90.0%)	16/17 (94.1%)	23/25 (92.0%)	20/23 (87.0%)	42/45 (93.3%)	0.657	0.821
Handgrip preserved (decline ≤ 1 kg)	40/46 (87.0%)	9/10 (90.0%)	15/17 (88.2%)	20/25 (80.0%)	20/23 (87.0%)	39/45 (86.7%)	0.960	0.960
Myoprotective fat-loss phenotype	9/46 (19.6%)	0/10 (0.0%)	1/17 (5.9%)	12/25 (48.0%)	13/23 (56.5%)	0/45 (0.0%)	<0.0001	<0.0001

Note. Values are n/N (%). The full myoprotective phenotype required fat-mass reduction ≥ 5%, lean-mass preservation, and handgrip preservation. Global *p*-values for each response criterion were obtained using Pearson χ^2^ tests across the six treatment groups; because several cells had expected counts < 5, each χ^2^ *p*-value was corroborated by a Monte Carlo permutation test (10,000 permutations of treatment labels), and the permutation *p*-value is the one reported where the two differed. FDR q-values were calculated using Benjamini–Hochberg correction across the binary response criteria in this table. SU, sulfonylurea; DPP-4i, dipeptidyl peptidase-4 inhibitor; SGLT2i, sodium-glucose cotransporter-2 inhibitor; GLP-1 RA, glucagon-like peptide-1 receptor agonist. All treatment groups received metformin as background therapy; group labels denote the added agent.

**Table 2:** Mutually exclusive weight-loss quality phenotypes by treatment group.

Treatment Group	n	Myoprotective Fat-Loss	Mixed Fat-Loss with Muscle/Function Cost	Low Fat-Loss But Functionally Stable	Non-Fat-Loss/ Functional-Risk
Metformin	46	9 (19.6%)	5 (10.9%)	21 (45.7%)	11 (23.9%)
Metformin + SU	10	0 (0.0%)	0 (0.0%)	2 (20.0%)	8 (80.0%)
Metformin + DPP-4i	17	1 (5.9%)	1 (5.9%)	7 (41.2%)	8 (47.1%)
Metformin + SGLT2i	25	12 (48.0%)	7 (28.0%)	6 (24.0%)	0 (0.0%)
Metformin + GLP-1 RA	23	13 (56.5%)	10 (43.5%)	0 (0.0%)	0 (0.0%)
Metformin + insulin	45	0 (0.0%)	0 (0.0%)	6 (13.3%)	39 (86.7%)
Overall	166	35 (21.1%)	23 (13.9%)	42 (25.3%)	66 (39.8%)

Note. Values are n (%). The global phenotype-by-treatment association was assessed using a Pearson χ^2^ statistic on the 6 × 4 contingency table, with statistical significance determined by Monte Carlo permutation (10,000 permutations of treatment labels) because multiple cells were sparse; the permutation *p*-value is reported. All treatment groups received metformin as background therapy; group labels denote the added agent.

**Table 3:** Unadjusted 12-month changes by treatment group.

Outcome	Metformin	Metformin + SU	Metformin + DPP-4i	Metformin + SGLT2i	Metformin + GLP-1 RA	Metformin + Insulin	*p*	FDR q
Δ weight (kg)	−1.21 ± 1.34	1.60 ± 1.19	−0.37 ± 1.61	−3.27 ± 1.15	−6.61 ± 1.18	1.87 ± 1.33	<0.0001	<0.0001
Weight loss (%)	1.27 ± 1.42	−1.65 ± 1.20	0.35 ± 1.63	3.22 ± 1.25	6.48 ± 1.56	−1.92 ± 1.44	<0.0001	<0.0001
Δ fat mass (kg)	−1.20 ± 1.43	0.76 ± 1.32	−0.62 ± 1.65	−2.83 ± 0.85	−6.01 ± 1.58	1.36 ± 1.42	<0.0001	<0.0001
Fat-mass loss (%)	2.97 ± 3.43	−2.04 ± 3.60	1.21 ± 3.70	7.13 ± 2.69	14.76 ± 4.33	−3.36 ± 3.48	<0.0001	<0.0001
Δ fat mass (%) points	−0.71 ± 0.94	0.21 ± 0.95	−0.41 ± 1.00	−1.56 ± 0.71	−3.43 ± 1.07	0.57 ± 0.95	<0.0001	<0.0001
Δ lean mass (kg)	−0.32 ± 2.02	0.36 ± 2.44	0.31 ± 1.76	−0.51 ± 1.66	−0.78 ± 2.04	0.44 ± 1.68	0.069	0.108
Lean-mass loss (%)	0.48 ± 4.00	−0.08 ± 4.23	−0.73 ± 3.91	0.71 ± 2.73	1.42 ± 3.44	−0.78 ± 3.11	0.112	0.156
Δ skeletal muscle mass (kg)	0.42 ± 0.77	−0.04 ± 0.79	0.25 ± 0.63	0.41 ± 0.70	0.05 ± 0.67	0.32 ± 0.74	0.212	0.247
SMM loss (%)	−1.76 ± 3.27	0.04 ± 3.14	−1.16 ± 2.35	−1.46 ± 2.70	0.04 ± 2.68	−1.26 ± 2.97	0.134	0.171
Δ handgrip (kg)	0.63 ± 1.43	1.15 ± 1.46	0.59 ± 1.68	0.70 ± 1.85	0.81 ± 1.58	0.53 ± 1.57	0.797	0.797
Δ fat-to-lean ratio	−0.02 ± 0.04	0.02 ± 0.05	−0.02 ± 0.04	−0.05 ± 0.03	−0.09 ± 0.04	0.02 ± 0.04	<0.0001	<0.0001
Δ handgrip/weight	0.01 ± 0.02	0.01 ± 0.01	0.01 ± 0.02	0.02 ± 0.02	0.03 ± 0.02	−0.00 ± 0.02	<0.0001	<0.0001
Δ handgrip/SMM	0.01 ± 0.07	0.04 ± 0.06	0.01 ± 0.07	0.02 ± 0.08	0.04 ± 0.07	0.01 ± 0.07	0.581	0.625
Δ HbA1c (%)	−0.48 ± 0.24	−0.63 ± 0.22	−0.64 ± 0.23	−0.84 ± 0.23	−1.22 ± 0.19	−1.05 ± 0.26	<0.0001	<0.0001

Note. Values are mean ± SD. Delta values were calculated as 12-month value minus baseline value; for weight, fat mass, fat mass percentage, lean mass, skeletal muscle mass, and HbA1c, negative delta values indicate reductions over time. Positive loss percentages indicate reduction from baseline. Global *p*-values were obtained using Kruskal–Wallis tests across the six treatment groups, chosen because of unequal group sizes and potential non-normality of change distributions; FDR q-values were calculated using Benjamini–Hochberg correction across the unadjusted continuous comparisons in this table. All treatment groups received metformin as background therapy; group labels denote the added agent.

**Table 4:** Baseline characteristics by mutually exclusive weight-loss quality phenotype.

Variable	Myoprotective Fat-Loss	Mixed Fat-Loss with Muscle/Function Cost	Low Fat-Loss But Functionally Stable	Non-Fat-Loss/ Functional-Risk	*p*	FDR q
Age, years	56.40 ± 13.10	60.83 ± 12.79	61.36 ± 13.09	59.89 ± 12.82	0.380	0.986
Female sex	22/35 (62.9%)	12/23 (52.2%)	26/42 (61.9%)	39/66 (59.1%)	0.854	0.986
Diabetes duration, years	14.04 ± 8.29	16.47 ± 6.60	12.45 ± 7.09	13.25 ± 7.90	0.183	0.739
HbA1c baseline, %	8.80 ± 0.92	9.06 ± 0.94	8.95 ± 0.91	8.98 ± 1.03	0.690	0.986
Weight baseline, kg	103.97 ± 18.02	98.73 ± 18.97	101.69 ± 17.80	103.90 ± 17.32	0.716	0.986
BMI baseline, kg/m^2^	36.54 ± 4.88	33.90 ± 4.02	36.26 ± 4.26	36.92 ± 4.16	0.056	0.673
Fat mass baseline, %	40.23 ± 6.90	40.14 ± 6.32	40.39 ± 5.89	40.53 ± 5.96	0.925	0.986
Fat mass baseline, kg	41.70 ± 9.81	39.64 ± 10.00	41.02 ± 9.07	41.94 ± 8.76	0.746	0.986
Lean mass baseline, kg	57.96 ± 14.03	55.70 ± 13.64	56.81 ± 12.39	58.03 ± 13.30	0.857	0.986
Skeletal muscle mass baseline, kg	28.04 ± 8.67	27.48 ± 8.43	27.60 ± 7.51	28.34 ± 7.90	0.986	0.986
Handgrip baseline, kg	30.37 ± 11.43	31.71 ± 10.66	30.67 ± 9.30	30.85 ± 9.73	0.830	0.986
hs-CRP baseline	5.16 ± 3.16	4.74 ± 3.03	6.06 ± 3.28	5.36 ± 2.75	0.185	0.739

Note. Continuous variables are mean ± SD; categorical variables are n/N (%). *p*-values for continuous variables were obtained using Kruskal–Wallis tests across the four weight-loss quality phenotypes; *p*-values for categorical variables were obtained using Pearson χ^2^ tests, with Fisher’s exact test substituted where any expected cell count was <5. FDR q-values were calculated using Benjamini–Hochberg correction across all baseline comparisons in this table.

**Table 5:** Adjusted treatment contrasts for selected 12-month body-composition and functional outcomes.

Outcome	Contrast vs. Metformin	β (95% CI)	SE (HC3)	*p*	FDR q
Fat mass at 12 m (kg)	Metformin + SU	1.942 (0.939 to 2.945)	0.512	<0.001	<0.001
Fat mass at 12 m (kg)	Metformin + DPP-4i	0.726 (−0.215 to 1.668)	0.480	0.130	0.226
Fat mass at 12 m (kg)	Metformin + SGLT2i	−1.650 (−2.240 to −1.060)	0.301	<0.0001	<0.0001
Fat mass at 12 m (kg)	Metformin + GLP-1 RA	−4.689 (−5.462 to −3.916)	0.394	<0.0001	<0.0001
Fat mass at 12 m (kg)	Metformin + insulin	2.635 (2.021 to 3.250)	0.314	<0.0001	<0.0001
Fat mass % at 12 m	Metformin + SU	0.943 (0.234 to 1.653)	0.362	0.009	0.024
Fat mass % at 12 m	Metformin + DPP-4i	0.206 (−0.408 to 0.820)	0.313	0.510	0.656
Fat mass % at 12 m	Metformin + SGLT2i	−0.901 (−1.317 to −0.484)	0.213	<0.0001	<0.0001
Fat mass % at 12 m	Metformin + GLP-1 RA	−2.731 (−3.266 to −2.195)	0.273	<0.0001	<0.0001
Fat mass % at 12 m	Metformin + insulin	1.318 (0.908 to 1.729)	0.210	<0.0001	<0.0001
Lean mass at 12 m (kg)	Metformin + SU	0.824 (−0.957 to 2.605)	0.909	0.365	0.547
Lean mass at 12 m (kg)	Metformin + DPP-4i	0.541 (−0.590 to 1.671)	0.577	0.349	0.547
Lean mass at 12 m (kg)	Metformin + SGLT2i	−0.150 (−1.064 to 0.763)	0.466	0.747	0.841
Lean mass at 12 m (kg)	Metformin + GLP-1 RA	−0.401 (−1.446 to 0.645)	0.533	0.452	0.651
Lean mass at 12 m (kg)	Metformin + insulin	0.744 (−0.034 to 1.522)	0.397	0.061	0.130
Skeletal muscle mass at 12 m (kg)	Metformin + SU	−0.501 (−1.088 to 0.085)	0.299	0.094	0.176
Skeletal muscle mass at 12 m (kg)	Metformin + DPP-4i	−0.140 (−0.529 to 0.248)	0.198	0.480	0.651
Skeletal muscle mass at 12 m (kg)	Metformin + SGLT2i	−0.025 (−0.386 to 0.336)	0.184	0.891	0.911
Skeletal muscle mass at 12 m (kg)	Metformin + GLP-1 RA	−0.386 (−0.766 to −0.005)	0.194	0.047	0.117
Skeletal muscle mass at 12 m (kg)	Metformin + insulin	−0.081 (−0.414 to 0.251)	0.169	0.631	0.775
Handgrip at 12 m (kg)	Metformin + SU	0.708 (−0.373 to 1.789)	0.551	0.199	0.332
Handgrip at 12 m (kg)	Metformin + DPP-4i	0.199 (−0.743 to 1.142)	0.481	0.678	0.803
Handgrip at 12 m (kg)	Metformin + SGLT2i	0.120 (−0.684 to 0.925)	0.410	0.769	0.844
Handgrip at 12 m (kg)	Metformin + GLP-1 RA	0.302 (−0.558 to 1.161)	0.439	0.492	0.651
Handgrip at 12 m (kg)	Metformin + insulin	−0.066 (−0.714 to 0.582)	0.331	0.842	0.902
Fat-to-lean ratio at 12 m	Metformin + SU	0.037 (−0.001 to 0.076)	0.020	0.059	0.130
Fat-to-lean ratio at 12 m	Metformin + DPP-4i	0.004 (−0.019 to 0.028)	0.012	0.717	0.827
Fat-to-lean ratio at 12 m	Metformin + SGLT2i	−0.028 (−0.045 to −0.011)	0.009	0.001	0.003
Fat-to-lean ratio at 12 m	Metformin + GLP-1 RA	−0.074 (−0.095 to −0.054)	0.010	<0.0001	<0.0001
Fat-to-lean ratio at 12 m	Metformin + insulin	0.041 (0.024 to 0.059)	0.009	<0.0001	<0.0001
Handgrip/weight at 12 m ×100	Metformin + SU	−0.476 (−1.487 to 0.534)	0.516	0.356	0.547
Handgrip/weight at 12 m ×100	Metformin + DPP-4i	−0.075 (−1.036 to 0.885)	0.490	0.878	0.911
Handgrip/weight at 12 m ×100	Metformin + SGLT2i	0.758 (−0.098 to 1.613)	0.436	0.082	0.161
Handgrip/weight at 12 m ×100	Metformin + GLP-1 RA	2.081 (1.065 to 3.098)	0.519	<0.0001	<0.001
Handgrip/weight at 12 m ×100	Metformin + insulin	−0.992 (−1.686 to −0.298)	0.354	0.005	0.014
Handgrip/SMM at 12 m	Metformin + SU	0.034 (−0.010 to 0.078)	0.023	0.131	0.226
Handgrip/SMM at 12 m	Metformin + DPP-4i	0.015 (−0.027 to 0.058)	0.022	0.476	0.651
Handgrip/SMM at 12 m	Metformin + SGLT2i	0.009 (−0.028 to 0.047)	0.019	0.637	0.775
Handgrip/SMM at 12 m	Metformin + GLP-1 RA	0.035 (−0.003 to 0.073)	0.019	0.074	0.152
Handgrip/SMM at 12 m	Metformin + insulin	−0.001 (−0.032 to 0.030)	0.016	0.944	0.944

Note. Reference group: metformin monotherapy. Estimates are adjusted differences in the 12-month outcome derived from ANCOVA-type linear regression models; each model included treatment group, the baseline value of the corresponding outcome, age, sex, diabetes duration, and baseline BMI. *p*-values are Wald tests of the treatment-group coefficient computed with HC3 heteroskedasticity-consistent robust standard errors. FDR q-values were calculated using Benjamini–Hochberg correction across all adjusted treatment contrasts in this table. All treatment groups received metformin as background therapy; group labels denote the added agent.

**Table 6:** Spearman correlations between adiposity loss and muscle/function changes.

X	Y	n	Spearman Rho	*p*	FDR q
Fat-mass loss (%)	Lean-mass loss (%)	166	0.174	0.025	0.044
Fat-mass loss (%)	Δ handgrip (kg)	166	0.060	0.444	0.518
Weight loss (%)	Fat-mass loss (%)	166	0.948	<0.0001	<0.0001
Weight loss (%)	Lean-mass loss (%)	166	0.286	<0.001	<0.001
Δ fat-to-lean ratio	Δ handgrip (kg)	166	−0.131	0.093	0.130
Fat-mass loss (%)	Δ handgrip/weight	166	0.496	<0.0001	<0.0001
Fat-mass loss (%)	Δ handgrip/SMM	166	0.049	0.534	0.534

Note. Positive relative loss percentages indicate reduction from baseline. Delta handgrip values were calculated as 12-month handgrip minus baseline handgrip. Coefficients are Spearman rank correlations (rho); *p*-values are the corresponding asymptotic t-approximation tests of rho = 0. FDR q-values were calculated using Benjamini–Hochberg correction across the correlation analyses in this table.

## Data Availability

The data that support the findings of this study are available from the corresponding author upon reasonable request.

## Abbreviations

The following abbreviations are used in this manuscript:

12 m	12 months
ANCOVA	Analysis of covariance
BIA	Bioelectrical impedance analysis
BMI	Body mass index
CI	Confidence interval
DPP-4	Dipeptidyl peptidase-4
DPP-4i	Dipeptidyl peptidase-4 inhibitor
FDR	False-discovery rate
GLP-1	Glucagon-like peptide-1
GLP-1 RA	Glucagon-like peptide-1 receptor agonist
HbA1c	Glycated hemoglobin
HC3	Heteroskedasticity-consistent type 3
HOMA-IR	Homeostatic Model Assessment of Insulin Resistance
hs-CRP	High-sensitivity C-reactive protein
SD	Standard deviation
SE	Standard error
SGLT2	Sodium-glucose cotransporter-2
SGLT2i	Sodium-glucose cotransporter-2 inhibitor
SMM	Skeletal muscle mass
STROBE	Strengthening the Reporting of Observational Studies in Epidemiology
SU	Sulfonylurea
T2DM	Type 2 diabetes mellitus
χ^2^	Chi-square statistic
