# Correction: Bujdei-Tebeică et al. Myoprotective Fat-Loss Phenotypes in Obesity-Associated Type 2 Diabetes: A 12-Month Real-World Cohort Study of Metformin-Based Treatment Regimens. *Clin. Pract.* 2026, *16*, 141

**DOI:** 10.3390/clinpract16100191

**Published:** 2026-10-13

**Authors:** Ioana Bujdei-Tebeică, Anca Mihaela Pantea-Stoian, Doina Andrada Mihai, Simona Diana Ștefan, Cristian Serafinceanu

**Affiliations:** 1 Department of Diabetes, Nutrition and Metabolic Diseases, Carol Davila University of Medicine and Pharmacy, 050474 Bucharest, Romania; 2 National Institute of Diabetes, Nutrition and Metabolic Diseases N.C. Paulescu, 020475 Bucharest, Romania

## Missing Acknowledgments

In the original publication [1], the Acknowledgments were not included.

**Acknowledgments:** Article Processing Charge of this paper was supported by Carol Davila University of Medicine and Pharmacy, through the institutional program Publish not Perish.

The authors state that the scientific conclusions are unaffected. This correction was approved by the Academic Editor. The original publication has also been updated.
